# Gene Expression and Prognostic Value of NADPH Oxidase Enzymes in Breast Cancer

**DOI:** 10.3390/ijms25063464

**Published:** 2024-03-19

**Authors:** Andressa de Vasconcelos e Souza, Caroline Coelho de Faria, Leonardo Matta Pereira, Andrea Claudia Freitas Ferreira, Pedro Henrique Monteiro Torres, Rodrigo Soares Fortunato

**Affiliations:** Instituto de Biofísica Carlos Chagas Filho, Universidade Federal do Rio de Janeiro, Rio de Janeiro 21941-971, Brazil; andvasco.celos@gmail.com (A.d.V.e.S.); carolinefaria@biof.ufrj.br (C.C.d.F.); leonardo.matta@helmholtz-munich.de (L.M.P.); deiaclau@biof.ufrj.br (A.C.F.F.); monteirotorres@biof.ufrj.br (P.H.M.T.)

**Keywords:** breast cancer, NADPH oxidases, reactive oxygen species, tumor aggressiveness

## Abstract

NADPH oxidase enzymes (NOX) are involved in all stages of carcinogenesis, but their expression levels and prognostic value in breast cancer (BC) remain unclear. Thus, we aimed to assess the expression and prognostic value of NOX enzymes in BC samples using online databases. For this, mRNA expression from 290 normal breast tissue samples and 1904 BC samples obtained from studies on cBioPortal, Kaplan–Meier Plotter, and The Human Protein Atlas were analyzed. We found higher levels of NOX2, NOX4, and Dual oxidase 1 (DUOX1) in normal breast tissue. NOX1, NOX2, and NOX4 exhibited higher expression in BC, except for the basal subtype, where NOX4 expression was lower. DUOX1 mRNA levels were lower in all BC subtypes. NOX2, NOX4, and NOX5 mRNA levels increased with tumor progression stages, while NOX1 and DUOX1 expression decreased in more advanced stages. Moreover, patients with low expression of NOX1, NOX4, and DUOX1 had lower survival rates than those with high expression of these enzymes. In conclusion, our data suggest an overexpression of NOX enzymes in breast cancer, with certain isoforms showing a positive correlation with tumor progression.

## 1. Introduction

Breast cancer (BC) is the second most commonly diagnosed cancer and the most frequent malignancy in women worldwide. In 2018, an estimated 2.1 million new diagnoses and approximately 626,679 BC-related deaths have occurred [1]. Due to the inherent molecular and clinical heterogeneity, BC is associated with diverse risk factors, etiologies, treatment effectiveness, and prognosis [2,3,4,5]. Most studies profiling gene expression signatures have classified BC into five major molecular subtypes: luminal A (estrogen receptor (ER)-positive, progesterone receptor (PR)-positive, human epidermal growth factor receptor 2 (HER2)-negative), luminal B (ER-positive, PR-positive, HER2-positive), HER2-enriched (ER-negative, PR- negative, HER2-positive), basal-like or triple-negative (ER-negative, PR-negative, HER2-negative), and normal breast-like tumors (unclassified) [6,7]. More recently, the claudin-low subtype has been identified and characterized as negative for ER, PR, and HER2 expression, but with low expression of Ki67 and high expression of epithelial–mesenchymal transition (EMT)-related genes, which distinguishes this subtype from the basal-like [8].

The mammalian nicotinamide adenine dinucleotide phosphate (NADPH) oxidases (NOX) are transmembrane proteins that carry electrons across biological membranes, reducing molecular oxygen (O_2_) to superoxide anion (O_2_^•−^) or hydrogen peroxide (H_2_O_2_). The NOX enzymes family contains seven members, which are NOX1-5 and Dual oxidases 1 and 2 (DUOX1-2). All isoforms exhibit six highly conserved transmembrane domains, one NADPH binding site in the C-terminal region, one FAD binding site, and two histidine-linked heme groups in the transmembrane domains III and IV. Additionally, NOX5 and DUOX 1-2 show an intracellular calcium-binding site that is closely related to their activation. Unlike all other sources of reactive oxygen species (ROS), NOX enzymes generate ROS as their main function [9].

Accumulated evidence in recent decades highlights the role of NOX-derived ROS in virtually all steps of carcinogenesis (initiation, promotion, and progression) of different organs/tissues. Furthermore, NOX enzymes have been suggested as a prospective target in novel therapeutic approaches [10,11]. Regarding BC, some studies have shown that NOX might be important players in this disease, although most of them were conducted under in vitro conditions utilizing cell lines, and the expression levels and prognostic value of NOX in patients remain elusive [12,13,14]. Thus, the aims of this study were first to assess the expression and prognostic value of individual NOX family members in the different BC molecular subtypes using online databases.

## 2. Results

### 2.1. Expression of NOX Family Genes in the Breast Tissue

**Figure 1:**
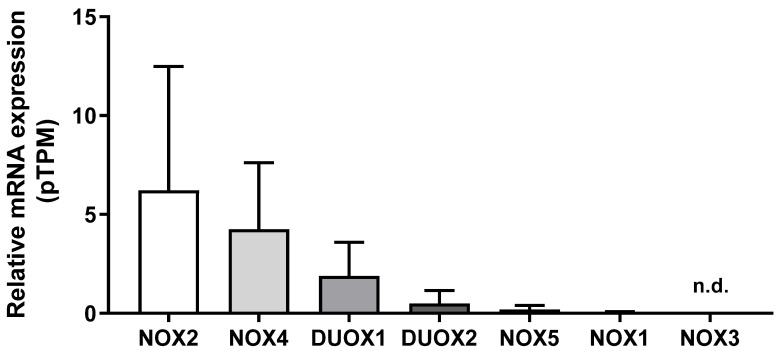
NADPH oxidases mRNA levels in the breast. Differences among NOX family mRNA levels in normal breast tissue. Data were obtained from the online platform Human Protein Atlas (https://www.proteinatlas.org, accessed on 2 July 2022). Results are represented as the mean protein-coding transcripts per million (pTPM) ± standard deviation (N = 290; n.d.: not detectable).

We first compared the mRNA levels of NOX family members in 290 samples of normal breast tissue using the HPA database. As shown in Figure 1, NOX2 mRNA levels were the most expressed, which was followed by NOX4, DUOX1, DUOX2, NOX5, and NOX1. NOX3 mRNA levels were undetectable. Therefore, we did not evaluate NOX3 in the next analysis.

### 2.2. Comparison of NOX mRNA Levels in Non-Tumoral and Breast Tumor Tissues

We next compared NOX mRNA levels between non-tumoral and breast tumor tissues. No data concerning DUOX2 expression were available, so this isoform was not analyzed. Due to breast cancer heterogeneity, we divided BC samples according to the five major molecular subtypes: luminal A, luminal B, HER2, basal, and claudin-low.

As demonstrated in Table 1, we observed that NOX1 mRNA levels were higher in the HER2 and basal subtypes when compared to the normal group. Similarly, the mRNA levels for NOX2 showed an increase in the HER2 and basal subtypes, in addition to claudin-low. Concerning NOX4 mRNA levels, they were revealed to be high in the luminal A, HER2, and claudin-low subtypes in comparison to normal tissue, while in the basal subtype, NOX4 mRNA levels were decreased. There were no statistically significant differences in NOX5 expression in any of the tumor subtypes when compared to the normal group. In relation to DUOX1, we detected a decrease in the mRNA levels in the luminal A, luminal B, HER2, and claudin-low subtypes when compared to the control.

**Figure 2:**
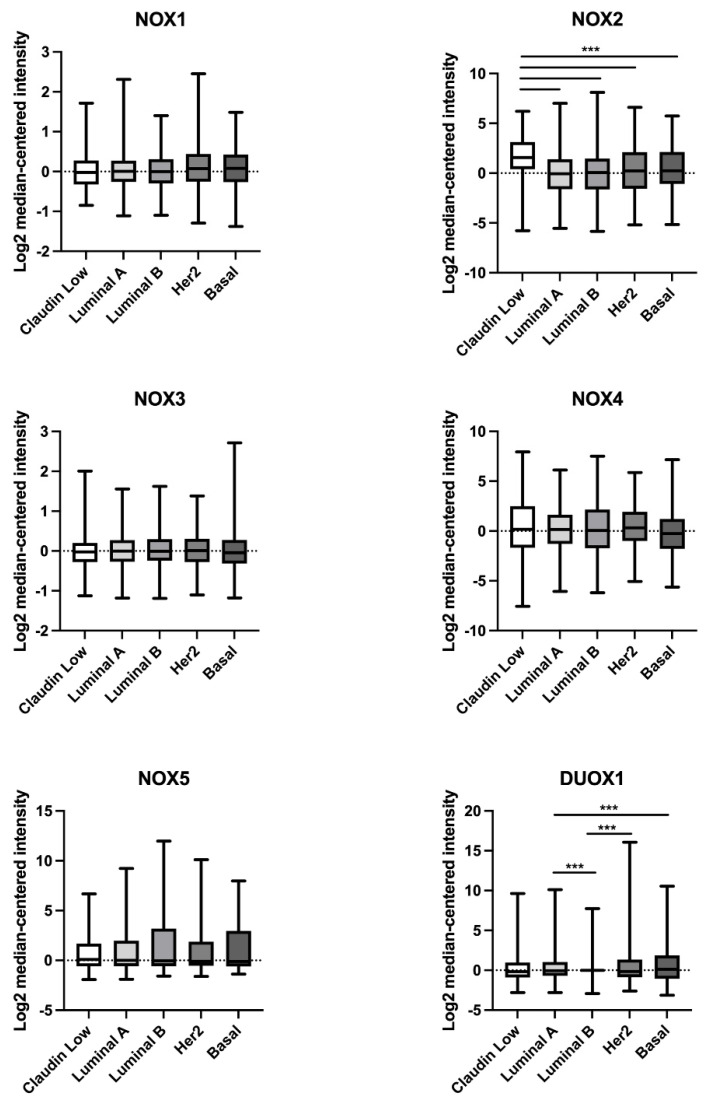
Comparison of NOX1, NOX2, NOX3, NOX4, NOX5, and DUOX1 mRNA levels in different subtypes of breast tumor tissues. Data were obtained from the cBioPortal online platform (http://www.cbioportal.org/, accessed on 3 July 2022) (N = 1904). Claudin-low (N = 199); luminal A (N = 679); luminal B (N = 461); HER2 (N = 220); basal (N = 199). *** *p* < 0.001.

As demonstrated in Figure 2, NOX2 mRNA levels were higher in the claudin-low BC subtype in comparison to the luminal A, luminal B, Her2, and basal subtypes. Furthermore, DUOX1 mRNA levels were lower in the luminal B BC subtype when compared to luminal A and Her2, and in luminal A in comparison to the basal subtype.

### 2.3. mRNA Expression of NOX Genes at Different Stages of Breast Tumor Development

Another parameter we have taken into consideration is BC staging. The American Joint Committee on Cancer (AJCC) TNM staging system takes into account three primary factors: tumor size (T), regional lymph node status (N), and distant metastases (M). These factors are used to determine an overall stage, which ranges from 0 to 4. Stage 0 is referred to as “carcinoma in situ”, indicating that the cancer remains confined to the primary layer of cells where it originated and has not spread. In this context, stage 1 BC is anatomically defined as a tumor smaller than 2 cm in size and shows no involvement of lymph nodes. On the other hand, stage 4 represents a state of advanced disease with distant metastases [4,15]. 

**Figure 3:**
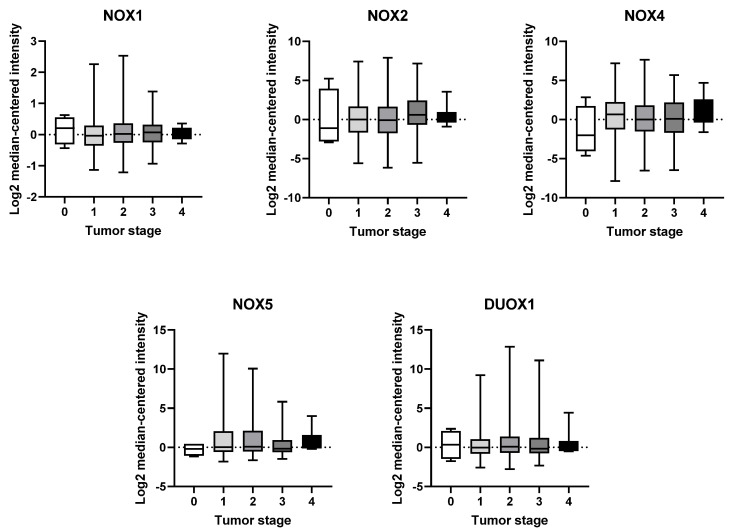
mRNA expression of NOX family at different stages of breast tumor. NOX1, NOX2, NOX4, NOX5, and DUOX1 were evaluated across tumor staging of breast tissues. Data were obtained from the cBioPortal online platform (http://www.cbioportal.org/, accessed on 3 July 2022) (N = 1904).

Based on our analysis, we have not observed any significant variations in the expression of any NOX isoform across the different tumor stages (Figure 3).

### 2.4. Survival Analysis of NOX Members in BC Patients

**Figure 4:**
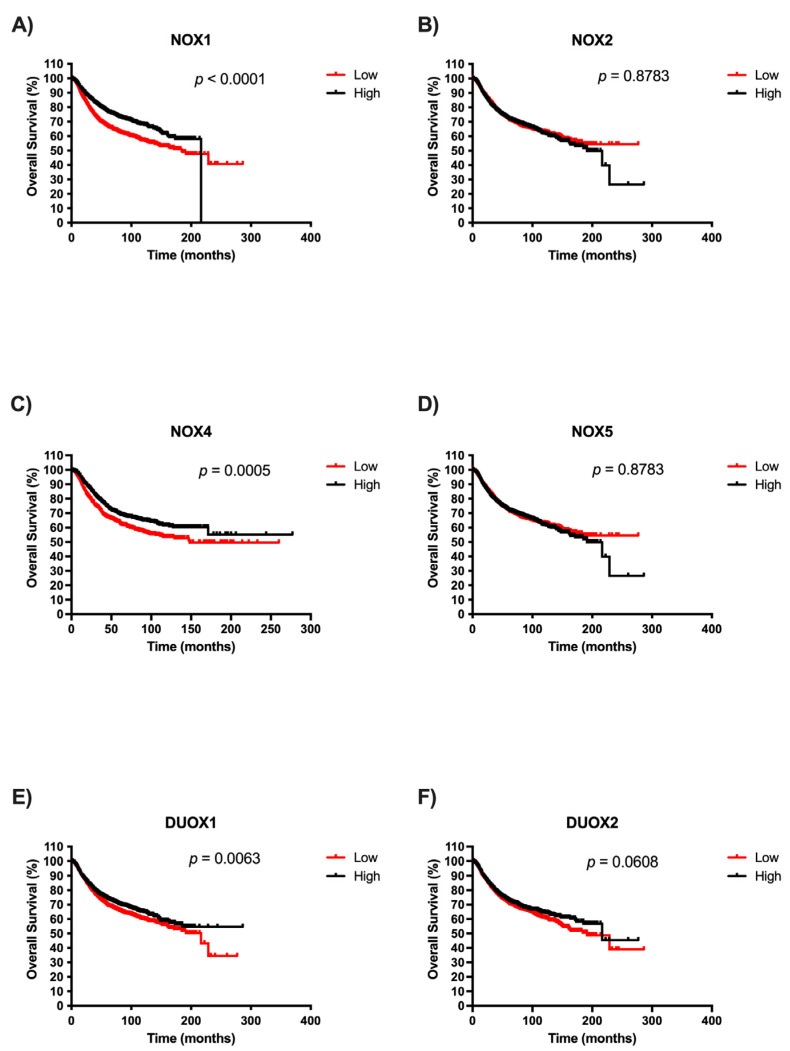
Survival analysis according to NOX family gene expression in BC patients. Comparison between the survival of BC patients with high or low expression of NOX1 (**A**), NOX2 (**B**), NOX4 (**C**), NOX5 (**D**), DUOX1 (**E**), and DUOX2 (**F**) in tumor breast tissues using Kaplan–Meier plotter analysis.

Afterwards, we examined the potential prognostic values of NOX isoforms in BC. The survival curves are demonstrated in Figure 4. It is important to highlight that there were no patient survival data related to high NOX1 expression after 200 months. We observed that patients with low expression of NOX1, NOX4, and DUOX1 exhibited significantly unfavorable prognoses in BC when compared with patients with high expression of those NOXs (Figure 4A, Figure 4C, and Figure 4E, respectively). Moreover, NOX2, NOX5, and DUOX2 expression were not associated with overall survival in BC (Figure 4B, Figure 4D, and Figure 4F, respectively).

### 2.5. NOX Gene Expression in Relation to Estrogen Receptor Status in Breast Cancer Samples

**Figure 5:**
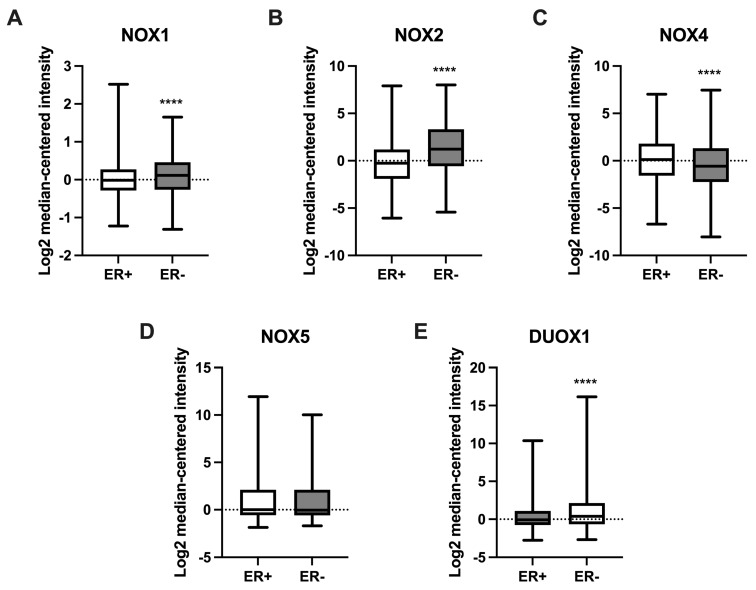
Expression of NOX family genes in relation to estrogen receptor (ER) status in BC samples. mRNA levels of NOX1 (**A**), NOX2 (**B**), NOX4 (**C**), NOX5 (**D**), and DUOX1 (**E**) in relation to the presence (ER+) or absence (ER-) of ER in tumor breast tissues. Data were obtained from the cBioPortal online platform (http://www.cbioportal.org/, accessed on 3 July 2022). N = 1904. **** *p* < 0.0001.

We further compared NOX mRNA levels between BC tissues that were positive or negative for the presence of estrogen receptors (ERs). As shown in Figure 5, NOX1 (Figure 5A), NOX2 (Figure 5B), and DUOX1 (Figure 5E) were higher in tissues that were ER-negative when compared to the ER-positive group. On the other hand, NOX4 expression (Figure 5C) was lower in ER-negative tissues in comparison to those with ER-positive status.

## 3. Discussion

Here, we show that NOX2 was the most expressed NOX isoform in normal tissue. NOX2 was originally the first identified NOX. It is highly expressed in leukocytes, where it has an essential role in phagocytic oxidative burst and host defense [11]. NOX2 expression was followed by NOX4, the only constitutively active isoform, which has been linked to physiological functions such as differentiation and memory formation [16,17]. Next, we observed DUOX1 and DUOX2 expression, respectively. Although DUOX2 plays an imperative role in hormone synthesis in the thyroid gland, DUOX1 has a diverse tissue distribution, but not a well-established physiological function [18,19]. It seems to be implicated in differentiation, wound responses, and host defense (reviewed in [20]). However, the role of DUOX1 and 2 in mammary tissue physiology is not known. Subsequently, we noticed NOX5, an isoform whose expression is typically found in the endothelium, spermatocytes, and lymphoid organs [21], and, finally, NOX1, which was mainly expressed in the colon and associated with gut immune modulation [22]. NOX3 mRNA levels were undetectable in our analysis, which might be due in part to its restricted distribution, found in high levels, for instance, in the inner ear, participating in ontogenesis [23]. To the best of our knowledge, no other study has evaluated the expression levels of NOX enzymes in normal human breast tissue.

Subsequently, we evaluated the expression levels of each NOX isoform by comparing the normal tissue to the different BC molecular subtypes. In a time in which personalized medicine is moving toward even more individualized treatment, the identification of new biomarkers among the subtypes should facilitate management planning. We found that the luminal A subtype had higher levels of NOX4 mRNA and lower levels of DUOX1. This subtype is low-grade, associated with a better prognosis, and tends to grow slowly [24]. In the luminal B subtype, which is known to grow slightly faster than luminal A cancers and to show worse recurrence-free survival at 5 years and 10 years [25], we observed a significant decrease in DUOX1 expression. In line with these findings, the HER2 subtype presented higher expression levels of NOX1, NOX2, and NOX4, but lower levels of DUOX1. The HER2 BC subtype was characterized as growing faster than luminal cancers and having a worse prognosis. Interestingly, these three subtypes exhibited low levels of DUOX1 expression. Data from our group demonstrated that DUOX1 was downregulated in BC cell lines and tissues. Moreover, non-tumor breast cells silenced for DUOX1 had a higher proliferation rate, as well as less adherence and migratory capacity when compared to control cells, suggesting that DUOX1 may play a tumor-suppressive role [26], which can be disrupted in luminal A, B, and HER2 BC subtypes according to our analysis.

Furthermore, in basal-like, also called triple-negative and considered the most aggressive and resistant BC subtype, NOX1 and NOX2 expression were significantly higher, in contrast to NOX4, which was diminished when compared to non-tumoral tissue. Some studies concerning NOX enzymes and BC have shown an upregulation of NOX1 in tumor tissues compared to normal tissues [27,28]. Desouki et al. (2005) demonstrated that NOX1 is highly expressed in breast tumors (86%). The authors also showed crosstalk between the mitochondria and NOX1 controlling redox signaling, and suggested that a disruption of this pathway could be involved in breast tumorigenesis [27]. Another study has proposed that the activation of the G protein-coupled receptor (GPCR) leukotriene receptor BLT2 promotes cell survival through the induction of NOX1- and NOX4-derived ROS in an ERK- and AKT-dependent mechanism in BC cell lines [29]. In addition, activation of the RAS-Erk1/2-NOX1 pathway was observed in a population of BC stem-like cells exposed to low concentrations of combined carcinogens, with an important function in the maintenance of increased cell proliferation [30].

NOX2 and NOX4 expression levels were higher in the claudin-low BC subtype, which is marked by aggressiveness, resistance to treatment, and poor prognosis [2]. Moreover, we observed higher NOX2 levels in three other BC subtypes (HER2, basal, and claudin-low). NOX2 has been related to angiogenesis, immune suppression, metastasis, and pro-survival signals in tissues such as the lung, as well as in leukemia and melanoma cells [31,32,33,34,35]. NOX2 was found in lipid rafts (LRs) of BC cell lines, whereas the disruption of these structures resulted in downregulation of the enzyme [36]. Mukawera et al. demonstrated that IKKε overexpression in BC cells was dependent on NOX2, leading to increased proliferation. NOX4 expression was also upregulated in three BC subtypes (luminal A, HER2, and claudin-low). Overexpression of NOX4 in non-tumor breast cells has been associated with increased senescence, cellular transformation, and resistance to apoptosis, suggesting an oncogenic role of this enzyme. The same authors also showed that NOX4 was overexpressed in various BC cell lines and primary breast tumors [12]. Additionally, another group has suggested NOX4 as a key player in TGFβ-induced epithelial-to-mesenchymal transition and migration of non-tumor and metastatic human breast cells [13]. In conformity, a subsequent study reported the efficiency of attenuating metastasis in BC cells by targeting NOX4 [37]. Our findings, in conjunction with those exposed in the literature, reinforce the great relevance of NOX4 in BC initiation and development.

Although, herein, we observed no changes in NOX5 expression levels among the BC subtypes, previous reports have identified NOX5 upregulation in BC, but they analyzed a limited number of samples with no molecular subtype distinction [14,38]. More recently, leptin has emerged as a potential activator of NOX5-derived ROS production in human epithelial mammary cells, linking obesity-associated hyperleptinemia with mammary tumorigenesis [39].

Surprisingly, in the present study, we did not identify significant alterations in NOX expression over tumor stages in our analysis. However, we verified the presence of increased expression levels of NOX1, NOX2, and DUOX1 in ER- BC tissues, and significantly low levels of NOX4 in ER+. ER is one of the main prognostic biomarkers in BC. The lack of the receptor is associated with a more aggressive profile of the tumor [40]. There are few studies correlating NOX expression and tumor progression. Liu and colleagues revealed a greater expression of NOX1 according to tumor progression in lung cancer [41]. Moreover, high expression of NOX4 and NOX5 were correlated to poorer overall survival in patients with end-stage gastric cancer [42].

Some studies have proposed that the expression level of NOX enzymes can influence the overall survival of patients. Individuals with hepatocarcinoma seem to have better prognoses when high expression levels of NOX4 mRNA are found, while patients with high levels of NOX2 present poor prognoses [43]. Analysis of gastric tumors has correlated NOX2 overexpression with longer patient survival, in contrast with NOX4 overexpression, which is associated with poorer overall patient survival [44]. It has also been reported that the high expression of DUOXs is related to reduced mortality in thyroid cancer [45]. In our analysis, patients with lower NOX1, NOX4, and DUOX1 expression exhibited shorter survival compared to patients with higher levels. Although we have observed high levels of expression of these proteins in tumor tissues, the survival data suggest that the loss of these NOX may occur in more undifferentiated cells and, consequently, more aggressive tumors, which would explain the lower survival rates of patients.

In conclusion, our data shed light on the role of NOX isoforms in BC. We highlight here that NOX2 is the most expressed NOX isoform in normal breast tissue, whereas, in conjunction with NOX4, it displays high levels in different BC subtypes. In the face of the plurality of BC molecular and histological characteristics, taking NOX expression into account could add significant value to the design and management of new therapies. However, many studies are still needed to elucidate the role of each NOX member in the different types of cancer and their implications.

## 4. Materials and Methods

### 4.1. Gene Expression Profiles in Breast Tissue

To analyze the expression of NOX family genes in normal breast tissue, we used the database The Human Protein Atlas (HPA) (https://www.proteinatlas.org, accessed on 2 July 2022). Individual genes (NOX1, CYBB NOX2, NOX3, NOX4, NOX5, DUOX1, and DUOX2) were evaluated. All quantitative transcriptome data (RNA-Seq) were retrieved from the Cancer Genome Atlas (TCGA) portal. A total of 290 normal breast samples were analyzed. The data were downloaded and plotted using the Graphpad Prism 9 software (GraphPad Software, Inc., San Diego, CA, USA).

### 4.2. Analysis of Gene Expression in Breast Tumor Tissue

The evaluation of NOX mRNA levels in BC patients was achieved through the online analysis tool cBioPortal (http://www.cbioportal.org/, accessed on 3 July 2022). The expression of NOX mRNA levels in human BC and normal tissues was obtained using the METABRIC study [3,45], containing 1904 samples from patients who had their breast tissue samples collected. We compared NOX mRNA levels between the different subtypes of BC and the non-tumoral tissue, as well as the influence of estrogen receptor status.

### 4.3. Analysis of Patient Survival According to NOX Expression

The significance of the prognosis in BC patients with different levels of NOX isoforms was performed using the Kaplan–Meier database (http://kmplot.com/analysis/, accessed on 15 August 2022). The BC database was established using gene expression data and survival information from 876 patients. Individual genes (NOX1, CYBB NOX2, NOX4, NOX5, DUOX1, and DUOX2) were evaluated on the platform. The patient cases were divided into two groups according to the median expression of the gene (high vs. low expression). The survival of patients in both groups was then analyzed using the Kaplan–Meier survival graph. A *p*-value less than 0.05 was considered statistically significant. The data were downloaded as text, which was replotted.

### 4.4. Statistical Analysis

Normality was verified using the D’Agostino and Pearson test. The data were analyzed using ANOVA, except in the results with only 2 groups, in which the *t* test was applied. Log2 median-centered intensity was calculated by subtracting the median from the values of both groups. The results were expressed as the mean ± standard deviation.

## Figures and Tables

**Table 1:** Comparison of NOX1, NOX2, NOX4, and DUOX1 mRNA levels in non-tumoral and different subtypes of breast tumor tissues.

Gene	BC Subtype	Non-Tumor	Tumor	*p* Value
NOX1	Claudin-low	0.03836	−0.02103	0.1291
	Luminal A	−0.02086	0.00429	0.9626
	Luminal B	0.01854	−0.0031	0.2762
	HER2	−0.09212	0.07419	0.0059
	Basal	−0.09197	0.08255	0.011
NOX2	Claudin-low	−2.2	1.576	<0.0001
	Luminal A	0.1081	−0.06415	0.763
	Luminal B	−0.1862	0.05626	0.0888
	HER2	−0.1628	0.2237	0.0483
	Basal	−0.2893	0.24	0.001
NOX4	Claudin-low	−0.3601	0.1767	0.036
	Luminal A	−1.125	0.1626	<0.0001
	Luminal B	−0.3539	0.0578	0.0564
	HER2	−0.9915	0.3326	<0.0001
	Basal	0.2339	−0.2542	0.0298
DUOX1	Claudin-low	0.2405	−0.1769	0.0112
	Luminal A	0.3179	−0.04951	0.0154
	Luminal B	0.1411	−0.00134	0.0104
	HER2	0.3012	−0.1476	0.0329
	Basal	−0.2282	0.1196	0.0686

Data were obtained from the cBioPortal online platform (http://www.cbioportal.org/, accessed on 3 July 2022). Non-tumor (N = 140); claudin-low (N = 199); luminal A (N = 679); luminal B (N = 461); HER2 (N = 220); basal (N = 199). Results are expressed as Log2 median-centered intensity.

## Data Availability

Data is contained within the article.
